# Understanding co-polymerization in amyloid formation by direct observation of mixed oligomers†
†Electronic supplementary information (ESI) available. See DOI: 10.1039/c7sc00620a



**DOI:** 10.1039/c7sc00620a

**Published:** 2017-05-09

**Authors:** Lydia M. Young, Ling-Hsien Tu, Daniel P. Raleigh, Alison E. Ashcroft, Sheena E. Radford

**Affiliations:** a Astbury Centre for Structural Molecular Biology , School of Molecular and Cellular Biology , University of Leeds , Leeds LS2 9JT , UK . Email: s.e.radford@leeds.ac.uk; b Department of Chemistry , Stony Brook University , Stony Brook , New York 11794-3400 , USA; c Genomics Research Center , Academia Sinica , 128 Academia , Taipei 11529 , Taiwan

## Abstract

Although amyloid assembly *in vitro* is commonly investigated using single protein sequences, fibril formation *in vivo* can be more heterogeneous, involving co-assembly of proteins of different length, sequence and/or post-translational modifications. Emerging evidence suggests that co-polymerization can alter the rate and/or mechanism of aggregation and can contribute to pathogenicity. Electrospray ionization-ion mobility spectrometry-mass spectrometry (ESI-IMS-MS) is uniquely suited to the study of these heterogeneous ensembles. Here, ESI-IMS-MS combined with analysis of fibrillation rates using thioflavin T (ThT) fluorescence, is used to track the course of aggregation of variants of islet-amyloid polypeptide (IAPP) in isolation and in pairwise mixtures. We identify a sub-population of extended monomers as the key precursors of amyloid assembly, and reveal that the fastest aggregating sequence in peptide mixtures determines the lag time of fibrillation, despite being unable to cross-seed polymerization. The results demonstrate that co-polymerization of IAPP sequences radically alters the rate of amyloid assembly by altering the conformational properties of the mixed oligomers that form.

## Abstract 2

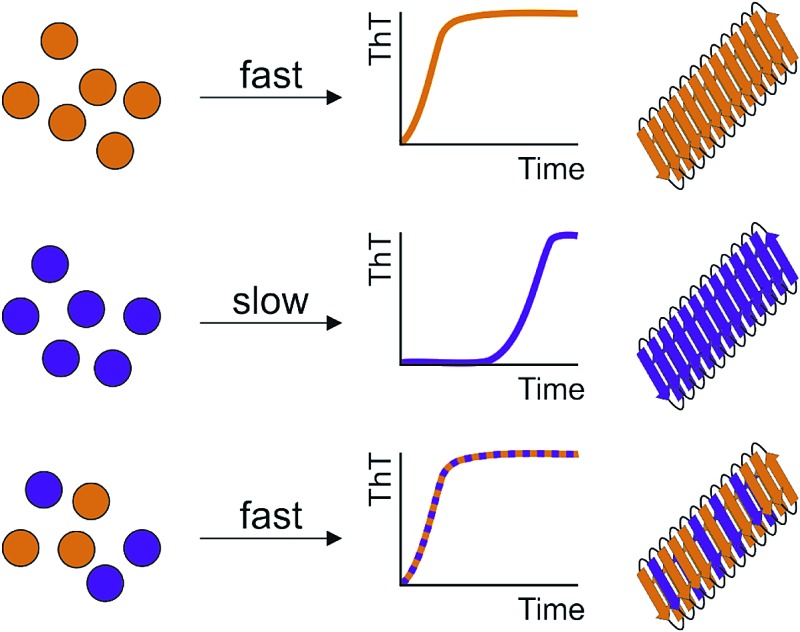
Co-assembly into hetero-oligomers controls the lag time of amylin assembly by a mechanism reminiscent of prions.

## Introduction

Amyloid disorders are characterized by the aggregation of proteins or peptides into amyloid fibrils.^1–3^ The ability to form amyloid is an inherent property of many polypeptide sequences,^4^ and denaturation or destabilization of the native state of a protein can increase its amyloidogenicity.^5,6^ Side-chain interactions and sequence similarity play crucial roles in amyloid assembly and in determining the ability of one protein sequence to seed polymerization and/or to co-polymerize with another. *In vitro*, amyloid fibrils are commonly assembled from a single protein sequence. However fibrils formed *in vivo* can contain multiple protein sequences, and the capacity to co-assemble may play a role in both the rate of aggregation and pathogenicity of amyloid deposition diseases.^7–16^

**Fig. 1:**
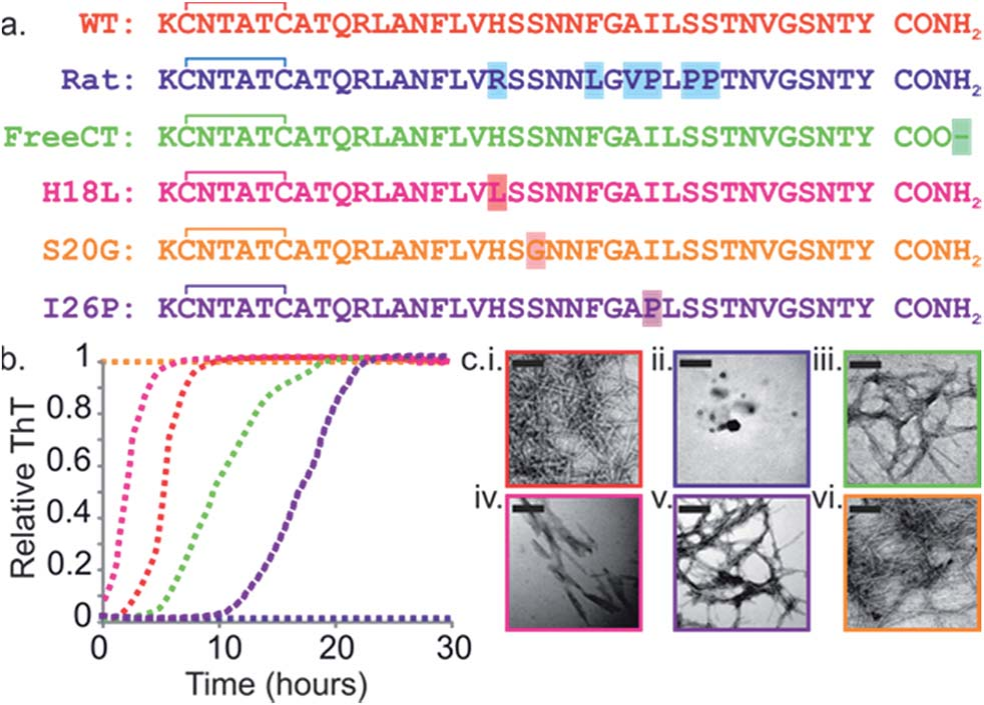
Sequence and amyloid formation of IAPP variants. (a) Primary sequence of the IAPP peptides studied here. Differences in sequence compared with WT are highlighted. (b) ThT fluorescence experiments (32 μM peptide, 25 °C, quiescent) in a 1 : 1 (v/v) mixture of 100 mM ammonium acetate: 100 mM ammonium bicarbonate, 1% (v/v) DMSO pH 7.4 show different lag times for different IAPP sequences (peptides are colored as in (a)). See also ESI Fig. 2.† (c) Representative negative stain TEM images after 5 days incubation (scale bar = 100 nm) for each sequence, colored as in (a).

Islet amyloid polypeptide (IAPP, also known as amylin), a peptide hormone produced by the β-cells of the pancreas,^17–19^ is intrinsically disordered, but forms amyloid in the pancreatic islets of Langerhans in type 2 diabetes mellitus (T2D) patients by an unknown mechanism.^17,20^ Wild type human IAPP (WT) is 37 residues in length, and contains a disulfide bond between Cys-2 and Cys-7, and an amidated C-terminus (Fig. 1a). The hormone is hydrophobic, but is cationic at physiological pH due to its free N-terminus, Lys-1, Arg-11 and His-18. Given that subtle alterations in the peptide sequence can result in dramatic differences in the rate of amyloid assembly of different IAPP variants (Fig. 1a and b), assessment of the ability of different sequences to co-polymerize has the potential to provide new insights into the origins and specificity of protein–protein interactions in amyloid assembly and the identification of species that govern the assembly rate. Indeed, previous *in vitro* studies using WT human and rat IAPP have demonstrated that the peptides are able to co-polymerize into mixed amyloid fibrils,^8,9,21^ with only a minor effect on the rate of assembly, despite the rat sequence being incapable of assembly into amyloid in isolation.^7,8^ Interestingly, a recent study on IAPP demonstrated that not all oligomers are toxic, suggestive that alteration in the conformational properties of oligomeric intermediates may alter the biological outcomes of the assembly process.^22^

Oligomers of IAPP have largely eluded detailed structural characterisation. Monomers through hexamers have been reported using ESI-IMS-MS^9,23^ and photochemical induced cross-linking.^22^ Abedini *et al.* used time-resolved toxicity assays to demonstrate that these early, low-order oligomers are responsible for the induction of reactive oxygen species and β-cell death.^22^ In contrast, several earlier studies had concluded that the size of the toxic species in IAPP amyloidosis is much larger than those reported here.^24–26^ For example, analytical ultracentrifugation studies failed to detect any oligomers containing <100 monomers, suggesting either that high order species dominate, or that smaller oligomers are unstable.^24^ Similarly, Ramamoorthy *et al.* used pulsed-field gradient-nuclear magnetic resonance (PFG NMR) to suggest that IAPP oligomers composed of <100 monomers form <1% of the total population of pre-fibrillar IAPP, such lowly populated oligomers are easily detectable using ESI-IMS-MS.^25^ Light-scattering has also been used to describe ‘intermediate-sized toxic amyloid particles’ of IAPP, containing anywhere between 25–6000 monomer units,^26^ while Gazit *et al.* described a cytotoxic, 90 kDa oligomer, corresponding to approximately 23 IAPP molecules.^27^ A study of cardiac amylin accumulation in obese T2DM patients reported that large amylin oligomers, >32 kDa in size, identified using western blots, contribute to heart failure, whilst smaller oligomers *i.e.* trimers and tetramers are reported to dominate in the early stages of amylin deposition in non-failing hearts.^28^

Co-polymerization of different amyloid sequences has been shown to occur in several amyloid systems (although not in islet amyloid in T2D), including β_2_-microglobulin and its truncated variant ΔN6 (proteins involved in dialysis-related amyloidosis),^8^ amyloid-β-peptide variants (Aβ40, Aβ42, Aβ43 and pyroglutamylated Aβ) associated with Alzheimer disease,^29–31^ as well as mixtures of Aβ40 and WT human IAPP.^32^ Heterogeneity can also occur by cross-seeding, a phenomenon which occurs when fibrils, (known as ‘seeds’), from one precursor sequence enhance fibrillation of a variant sequence, *via* templating of the precursor's structure. Seeded fibrils form at an increased rate compared with their unseeded counterparts and can be structurally distinct from fibrils formed *de novo*.^10,33,34^

Recent studies have suggested that the sequence determinants of cross-seeding and co-polymerization are distinct.^8,32,35^ Here, using variants of IAPP as a model system, we explore the ability of polypeptides with similar sequence, but different amyloid propensity, to influence amyloid assembly *via* conformational conversion, co-polymerization and/or cross-seeding. We show that population of a specific conformer (the most expanded monomeric species for the sequences studied) directly correlates with the lag time of IAPP assembly and discover a remarkable behavior of IAPP sequences in which the more rapidly assembling sequences accelerate aggregation of their less amyloid-prone counterparts, reminiscent of “prion-like” behavior. The requirements for cross-seeding are more stringent, however, with single point mutations precluding the ability of the sequences to interact.

## Experimental

### Sample preparation for MS

IAPP peptides were synthesized and purified as described previously.^36^ Lyophilized peptides were dissolved in dimethyl sulfoxide (DMSO) at a final peptide concentration of 3.2 mM. After 24 h incubation at 25 °C, stock solutions were diluted 100-fold into a solution containing a 1 : 1 mixture of 100 mM ammonium acetate and 100 mM ammonium bicarbonate, pH 7.4, to a final peptide concentration of 32 μM for MS analysis. The final concentration of DMSO was 1% (v/v). All samples were incubated at 25 °C in 96-well plates (Corning Costar 3915, Corning Life Sciences, Amsterdam, The Netherlands), without agitation.

### ESI-(IMS)-MS analysis

A Synapt HDMS quadrupole time-of-flight mass spectrometer (Micromass UK Ltd., Waters Corpn., Manchester, UK), equipped with a Triversa NanoMate (Advion Biosciences, Ithaca, NY, USA) automated nano-ESI interface, was used in this study. The instrument has a travelling-wave IMS device situated in-between the quadrupole and the time-of-flight analyzers, as described in detail elsewhere.^37^ Samples were analyzed by positive ionization nanoESI (nESI) with a capillary voltage of 1.7 kV and a nitrogen nebulizing gas pressure of 0.8 psi. The following instrumental parameters were used: cone voltage 30 V; source temperature 60 °C; backing pressure 2.0 mbar; ramped travelling wave height 7–20 V; travelling wave speed 400 m s^–1^; IMS nitrogen gas flow 20 mL min^–1^; IMS cell pressure 0.55 mbar. Data were processed by use of MassLynx v4.1 and Driftscope software supplied with the mass spectrometer. The *m*/*z* scale was calibrated with aq. CsI cluster ions. CCSs were estimated by use of an IMS-MS calibration.^38^ Calibration of the drift time cross-section function was achieved by analysis of the denatured proteins equine cytochrome c and horse heart myoglobin (10 μM in 50 : 40 : 10, v/v/v, acetonitrile, water, acetic acid^39^), whose CCS values had been pre-determined elsewhere by use of conventional ion mobility measurements.^39^ The collision cross-sectional areas (*Ω*) of the analytes were then obtained, after calibration, from their corrected drift times according to eqn (1).^39^
1
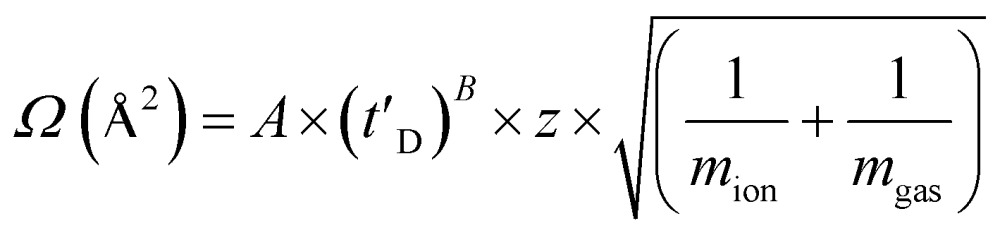



This step also includes an adjustment for the mass and charge of the protein ions: where *Ω* is the calibrated collision cross-section, *A* is the calibration determined constant, *t*′_D_ is the absolute drift time (corrected), *z* is the charge state of the ion, *m*_ion_ is the mass of the ion, and *m*_gas_ is the mass of the target gas used in the IMS cell. The exponential factor *B* is determined experimentally.^38^

### Thioflavin T fluorescence assays

Samples were prepared in a 96-well plate (Corning Costar 3915, Corning Life Sciences, Amsterdam, The Netherlands) sealed with clear sealing film (BMG Labtech, Aylesbury, Bucks, UK) and were incubated in a FLUOstar OPTIMA plate reader (BMG Labtech, Aylesbury, Bucks, UK) for 5 days at 25 °C without agitation. Samples had a volume of 100 μL containing 100 μM ThT and 32 μM peptide in a 1 : 1 (v/v) mixture of 100 mM ammonium acetate: 100 mM ammonium bicarbonate, pH 7.4, and a 1% (v/v) final concentration of DSMO. The ThT studies used excitation and emission filters of 430 and 485 nm, respectively. Aliquots of fibrils formed from pure peptide ThT experiments were used to seed other monomer solutions. The concentration of the seeds was 3.2 μM in the monomer units. The lag time was determined by extrapolating the elongation phase down to the intercept with the pre-transition base-line.

### Transmission electron microscopy (TEM)

TEM images of each 32 μM peptide solution were acquired after 5 days incubation at 25 °C on a JEM-1400 (JEOL Ltd., Tokyo, Japan) transmission electron microscope. Carbon grids were prepared by irradiating under UV light for 30 min and stained with 4% (w/v) uranyl acetate solution as described previously.^40^

### Fibril depolymerization

Mixed samples containing a 1 : 1 molar ratio of two variant peptides were prepared by diluting 3.2 mM stock solutions of each peptide in DMSO 100-fold into a 1 : 1 (v/v) mixture of 100 mM ammonium acetate: 100 mM ammonium bicarbonate, pH 7.4, to a final concentration of each peptide of 32 μM in 1% (v/v) DMSO. After 5 days of incubation at 25 °C, quiescent as for MS analysis, mixed samples were centrifuged in a Beckman ultracentrifuge at 300 000 g for 45 min. Pellets were depolymerized by incubation in 100% (v/v) hexafluoroisopropanol (HFIP) for 24 h (25 °C, 200 rpm). Samples were air-dried and then redissolved in 50 : 40 : 10 (v/v/v) acetonitrile/water/acetic acid and fibril constituent peptides were identified by ESI-MS.

## Results and discussion

### Tuning the rate of aggregation of IAPP by sequence variations

The rate of amyloid formation by IAPP *in vitro* is sequence dependent.^19,41–46^ WT IAPP forms thioflavin-T (ThT)-positive species after 2 h incubation at pH 7.4 under the conditions of these studies (Fig. 1b and c), Rat IAPP (Fig. 1a) does not aggregate into amyloid-like fibrils (using 32 μM peptide) (Fig. 1b and c), despite these peptides differing by only six residues.^9,47^ The single point mutants (H18L, S20G, and I26P) and a variant with a free carboxyl at the C-terminus (FreeCT) (Fig. 1a) exhibit altered rates of amyloid formation compared with WT.^48^ The missense S20G variant of the human IAPP gene is associated with a modest increase in the risk of T2D in certain Asian populations and has been shown to be more toxic to cultured cells.^49^ This peptide forms amyloid without a lag phase *in vitro* (Fig. 1b and c).^49^

The H18L variant has an increased rate of amyloid formation compared with WT, with no lag phase observed (Fig. 1b).^50^ FreeCT forms amyloid more slowly than WT (Fig. 1b).^50^ I26P, which has been shown previously to be a moderate inhibitor of WT amyloid formation,^22,42,51^ forms ThT-positive species with a lag phase ∼5 times longer than that of WT under the conditions employed here (Fig. 1b). The fibrils produced by the variants have a variety of morphologies (Fig. 1c) and, in the case of I26P, amorphous aggregates form along with fibrils (ESI Fig. 1†). These variants thus provide a set of similar sequences which span a wide range of aggregation rates and include peptides which form amyloid more rapidly than WT, as well as variants which assemble more slowly than WT or are incapable of forming amyloid when incubated alone.

### Analysis of monomers and oligomers of IAPP variants using ESI-IMS-MS

**Fig. 2:**
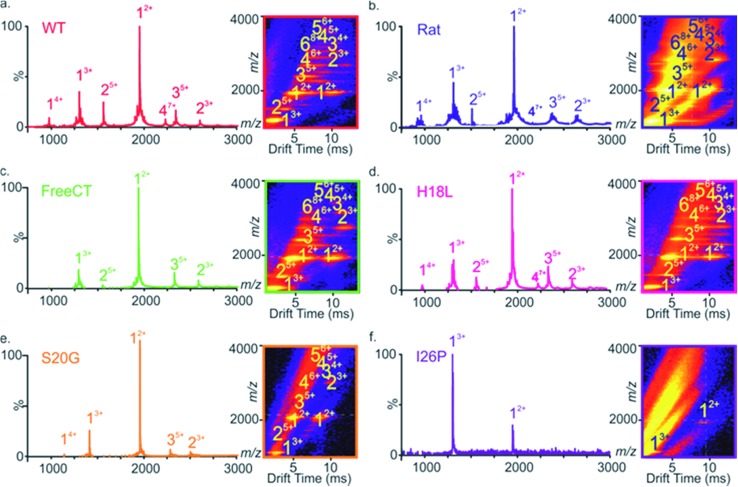
ESI-mass spectra of different IAPP variants. (a–f) ESI mass spectra of different IAPP variants show dominant 2+ and 3+ charge state ions of IAPP monomers (labeled 1) and minor amounts of dimers, trimers and tetramers (labeled 2, 3 and 4, respectively), except for the I26P variant for which only monomeric ions are observed. ESI-IMS-MS Driftscope plots (a–f inset) of the IAPP variants show oligomers present 2 min after diluting peptide monomers to a final peptide concentration of 32 μM, while only monomers of I26P are detected. All spectra were obtained in a 1 : 1 (v/v) mixture of 100 mM ammonium acetate: 100 mM ammonium bicarbonate, 1% (v/v) DMSO pH 7.4. ESI-IMS-MS Driftscope plots show IMS drift time *versus m*/*z versus* intensity (*z* = square root scale). Numbers above peaks denote oligomer order, with the positive charge state of the ions given as a superscript.

Electrospray ionization-ion mobility spectrometry-mass spectrometry (ESI-IMS-MS) is a powerful method for studying the conformations of intrinsically unstructured peptides and proteins, and has been used to analyze the self-assembly of several intrinsically disordered amyloid precursors, including Aβ,^32,52–54^ α-synuclein^55,56^ and IAPP.^9,23,57–59^ ESI-IMS-MS has the unique ability to identify transient, heterogeneous and lowly-populated intermediates that are co-populated during amyloid assembly and to determine the relative proportion of different conformational states that are co-populated, for example during the course of aggregation.^9,60^ The ESI mass spectrum of WT IAPP (Fig. 2a) shows predominantly monomer-related ions (*e.g.* 1^2+^ and 1^3+^), with traces of dimer through to hexamer, the oligomers being most readily observed using ESI-IMS-MS, consistent with previous results^9,23^ (Fig. 2a inset).

A similar distribution of monomer charge states and oligomeric species are observed using ESI-MS for the non-amyloidogenic Rat IAPP (Fig. 2b),^9^ as well as for variants that form amyloid more slowly (FreeCT – Fig. 2c), or more rapidly (H18L – Fig. 2d and S20G – Fig. 2e) than WT. The I26P variant, which forms amyloid most slowly (Fig. 2f), shows a significant alteration in the charge state distribution of monomer ions, with a change in the ratio of 3+ : 2+ monomer charge states from ∼1 : 3 (for WT and the other peptides examined) to ∼3 : 1. Similar alterations have been observed previously for WT IAPP in the presence of small molecule inhibitors^9,23^ and may reflect an alteration in the capacity to form amyloid-competent conformations. The three-dimensional Driftscope plot for I26P (Fig. 2f inset) displays predominantly monomeric peaks with little (or no) signal resulting from higher order oligomers, consistent with the low amyloid propensity of this variant.^48^ In the case of S20G, signals arising from oligomers are present (Fig. 2e inset), but are lower in intensity then those observed for WT and the other amyloid-prone variants, presumably due to the rapid consumption of oligomers into fibrillar structures which cannot be detected by ESI-MS or ESI-IMS-MS, as observed previously for WT under high ionic strength conditions.^9^

### Oligomer consumption is concomitant with assembly into amyloid

To investigate whether the presence of oligomers correlates with the rate of peptide assembly into amyloid fibrils, time course experiments were performed (ESI Fig. 3†). Oligomers were observed using ESI-IMS-MS at different points during aggregation and compared with the rate of fibril formation measured using ThT fluorescence assays. Under the conditions employed, the rate of formation of ThT-positive species observed is S20G > H18L > WT > FreeCT ≫ I26P (Fig. 1b). For S20G and I26P, the fastest and slowest aggregating variants, respectively, oligomer consumption over time is difficult to measure using ESI-IMS-MS. For S20G, this is likely due to oligomer consumption being too rapid, while for I26P the oligomers are of too low intensity to be detected by this method (Fig. 2e and f). It is clear, however, for WT, FreeCT and H18L that the rate of loss of oligomer signal intensity mirrors the length of the lag phase (ESI Fig. 3†), consistent with the oligomers being involved in amyloid assembly.^9^

### The population of expanded monomeric conformers correlates with amyloid propensity

**Fig. 3:**
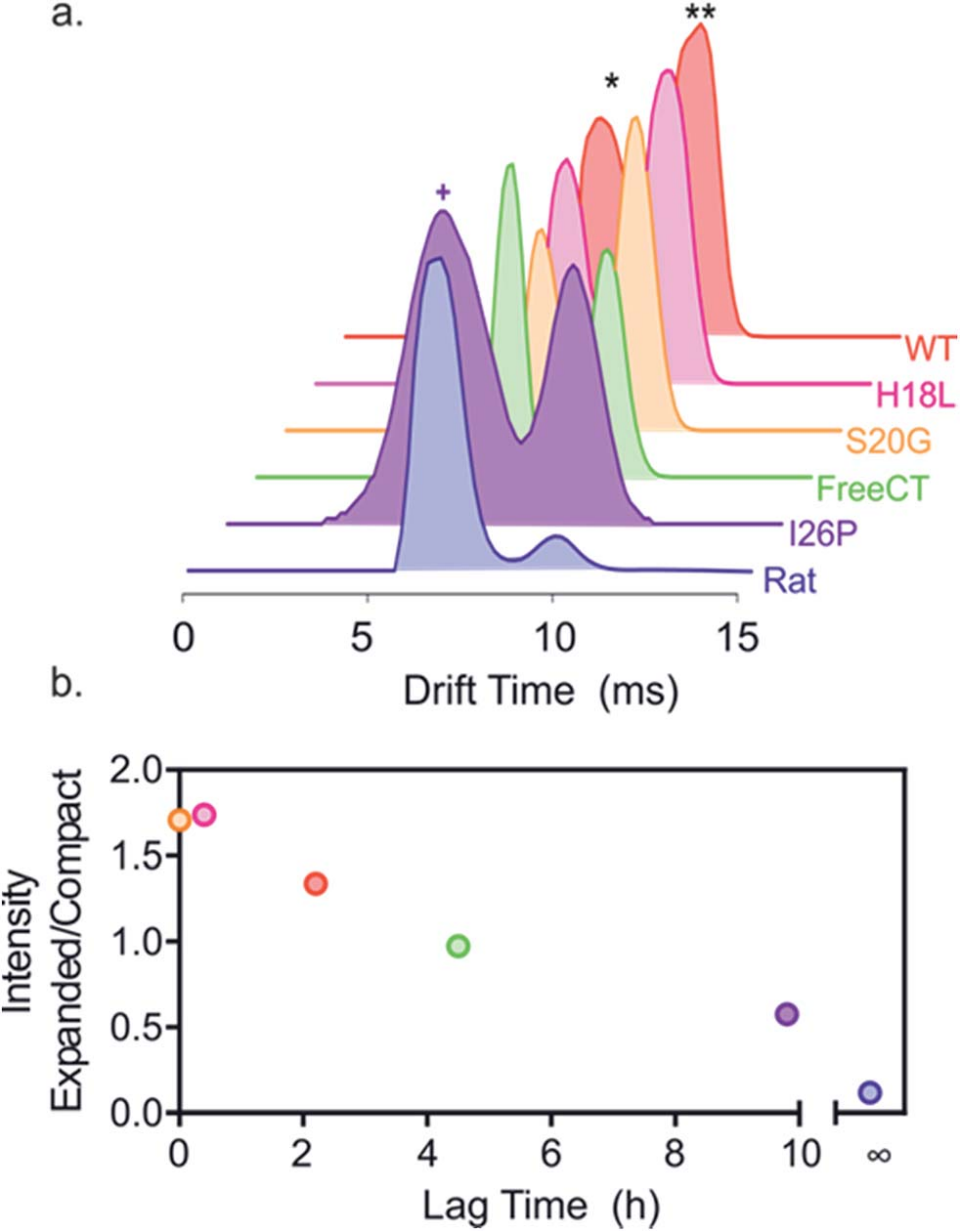
Different relative populations of IAPP monomer 2+ ions correlate with the lag time of assembly. (a) ESI-IMS-MS arrival time distributions show that 2+ monomer ions of each variant occupy two dominant conformers (* and **, highlighted for WT). I26P (purple) also occupies additional more compact conformers (purple +), not observed for the other peptides. Experimental CCSs of these 2+ monomers, measured using ESI-IMS-MS, are 4.4 and 5.8 nm^2^, respectively. The more compact I26P monomer conformation has a CCS of 4.1 nm^2^. The experimental error is ± 5% for all cross-sections measured using IMS-MS calibration.^38,61^ (b) Plot of relative area under peaks of compact/expanded monomeric conformers (drift times ∼6.0 and 9.8 ms, respectively) *vs.* the lag time of fibril assembly (colored as in (a)). The lag time of Rat IAPP is denoted as infinity (∞).

To investigate the effect of the amino acid substitutions on the distributions of different monomeric gas phase conformers of each IAPP variant, ESI-IMS-MS arrival time distributions (ATDs) of monomer ions of each peptide sequence were compared (Fig. 3). ESI-IMS-MS experiments allow separation of ions based upon their *m*/*z*, size and shape. Using a suitable calibration (Methods) the time taken for an ion to traverse the ion mobility cell enables determination of its collisional cross-section, and hence its relative compactness, with compact ions having higher mobility and shorter drift times with respect to expanded ions of the same mass and charge, which have lower mobility and longer drift times. Since ESI-IMS-MS does not require sample separation/purification, the relative population of conformers with distinct drift times can be individually quantified in complex mixtures and tracked *versus* time, offering advantages over solution methods in which only the weight average properties of species in rapid exchange can be obtained. Note that while ESI-IMS-MS enables conformers with different drift times to be identified, each peak identified by a distinct ATD could include an ensemble of structures of similar/identical drift times that are un-resolvable by IMS. While bulk solution cross-linking methods have detected the same oligomeric states of hIAPP, deduced by ESI-IMS-MS, they are unable to distinguish different conformers of a particular oligomer.^22^ For all variants, except I26P, doubly charged monomer ions occupy two distinct conformational ensembles with drift times ∼6.0 and 9.8 ms, but different relative abundances, as noted previously for WT and Rat IAPP.^9,57^ Importantly, the relative population of the different conformers correlates with the lag time of assembly, such that variants with the greater population of relatively expanded monomer 2+ ions form fibrils most rapidly, suggesting this conformer is the most amyloidogenic (Fig. 3b). Consistent with this, the non-amyloidogenic Rat IAPP lacks expanded monomer 2+ ions.^9^ Uniquely, 2+ ions of the variant I26P contain additional, more compact monomer species (drift times < 6 ms) (Fig. 3a). These conformers could be less able to assemble into fibrils than the other conformers observed, rationalizing the very slow assembly kinetics of this variant. Alternatively, they could be precursors of the amorphous aggregates which form concomitantly with fibrils for this variant (ESI Fig. 1†).

### All peptide variants are capable of co-assembly

**Fig. 4:**
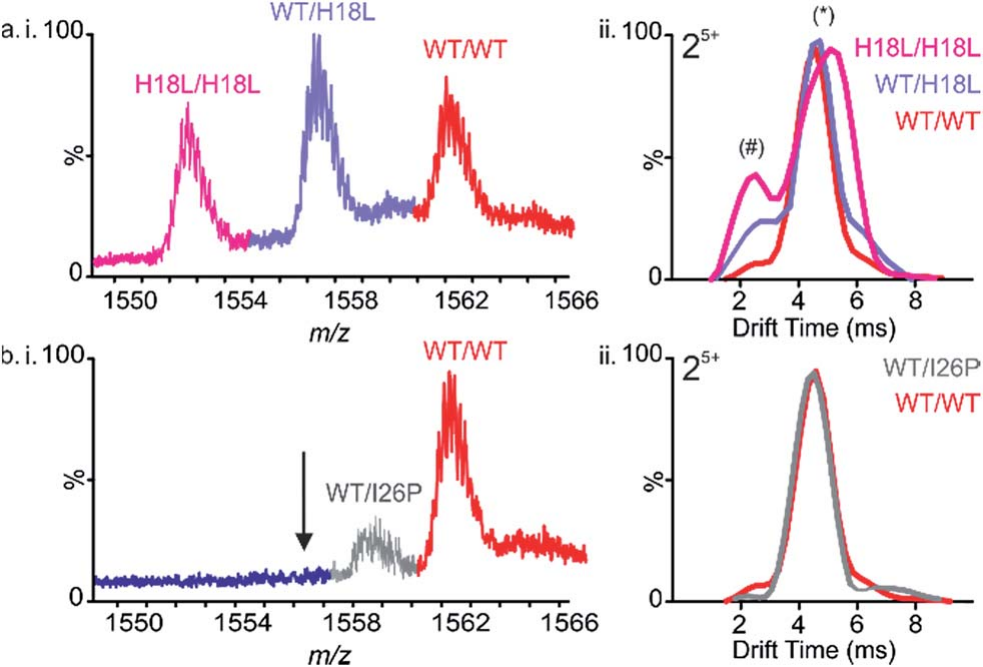
Mixing sequence variants of IAPP reveal different propensities to co-assemble. (a) (i) ESI mass spectrum of a 1 : 1 molar mixture of WT : H18L showing the presence of both homo- and hetero-dimers (5+ ions are shown). (ii) Arrival time distributions of homo- and hetero-dimers. Each dimer 5+ occupies one dominant conformer (*) with a small contribution of a second more compact conformer (#) with different relative intensities. (b) (i) ESI mass spectrum of a 1 : 1 mixture of WT : I26P showing the presence of WT 5+ homodimers and WT : I26P 5+ heterodimers but an absence of I26P 5+ homodimers. The position at which I26P 5+ homodimers would be observed is indicated with an arrow. (ii) Arrival time distributions of WT 5+ homo- and WT : I26P 5+ heterodimers.

**Fig. 5:**
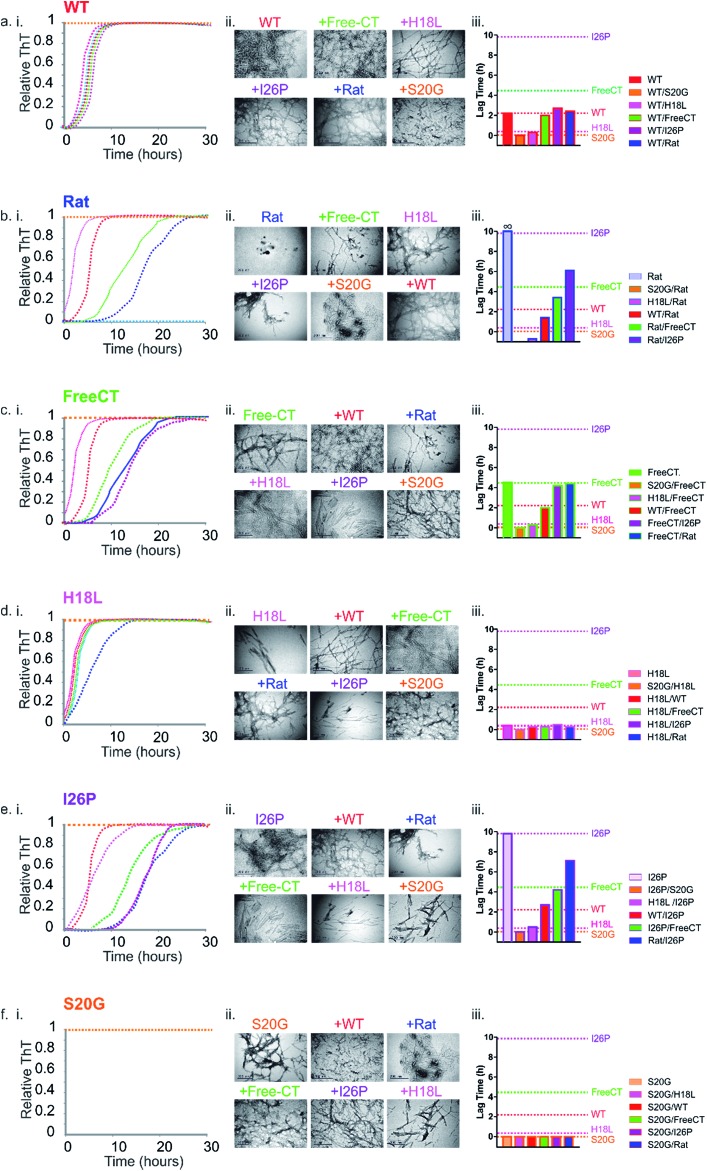
Prion-like behavior of IAPP peptide mixtures. (a–f) (i) ThT fluorescence intensity experiments following amyloid formation of IAPP sequence variants alone (indicated above each plot), as well as 1 : 1 mixtures of different peptide sequences (colored + WT (red)). +Rat (dark blue), +Free-CT (green), +H18L (red), +I26P (purple) and + S20G (orange). All assays were performed using 32 μM final peptide concentration, 25 °C, quiescent in a 1 : 1 (v/v) mixture of 100 mM ammonium acetate: 100 mM ammonium bicarbonate, 1% (v/v) DMSO, pH 7.4. (a) (i) WT and 1 : 1 mixtures of the different variants with WT, (b) (i) Rat and mixtures with Rat, (c) (i) Free-CT and mixtures with Free-CT, (d) (i) H18L and mixtures with H18L, (e) (i) I26P and mixtures with I26P and (f) (i) S20G and mixtures with S20G. ThT experiments for every sequence and mixture were repeated a minimum of three times, with at least triplicate measurements and representative traces are shown (see also ESI Fig. S6†). (a–f) (ii) Negative stain TEM images of each sample after 5 days in the same buffer (25 °C, quiescent) (scale bar = 200 nm). (a–f) (iii) Bar chart showing lag times from ThT fluorescence experiments of 1 : 1 mixtures of each sample. Errors on the lag time are +/– 10% on a single plate (ESI Fig. 6†).

To determine whether the IAPP variants are able to co-polymerize, aggregation of pairwise mixtures of IAPP peptides (1 : 1 molar ratio) was studied. ESI-IMS-MS was used to identify any hetero-oligomers formed and ThT analyses were performed to determine whether retardation or enhancement of the rate of IAPP assembly occurs in the peptide mixtures. As observed previously for WT and Rat IAPP,^9^ all peptide mixtures resulted in arrays of homo- and hetero-oligomers. In a 1 : 1 mixture of WT and H18L, for example, dimers are observed with *m*/*z* values corresponding to all-WT, all-H18L, and a mixture of WT and H18L monomer subunits in a ∼1 : 2 : 1 ratio, consistent with random mixing of the peptides (Fig. 4a(i)). Similarly, trimers are also observed at a ∼1 : 3 : 3 : 1 ratio of all-WT, WT/H18L/H18L, WT/WT/H18L and all H18L, again consistent with random mixing of the peptide sequences (ESI Fig. 4†). The ATDs of the all-WT, all-H18L and 1 : 1 WT : H18L dimers each occupy a dominant conformer (drift time ∼4.5 ms) along with a small population of a second, more compact conformer (drift time ∼2.5 ms) (Fig. 4a(ii)). Interestingly, the relative intensity of this second conformer is increased in the mixed dimer with respect to the WT homodimer, but is decreased with respect to the H18L homodimer, suggestive of a compromise in conformation in the hetero-dimeric species. We suggest that the occupation of a greater relative proportion of the more expanded conformer correlates with capacity to form amyloid at an increased rate. Thus because H18L homodimer, and the H18L-WT heterodimers, are relatively more expanded than the WT homodimer, these samples assemble into fibrils with a shorter lag time (Fig. 5a and d). Consistent with this notion, homo-trimers of WT result in only a single detectable species (arrival time ∼7.0 ms), while trimers of H18L and mixed WT/H18L trimers all show the presence of a second species (arrival time ∼8.6 ms), with the intensity of the latter peak increasing with the number of H18L monomers (∼2, 9, 14, 19% of the total ion intensity for all-WT, WT/WT/H18L, WT/H18L/H18L and all-H18L, respectively). By contrast, in the 1 : 1 mixture of WT and I26P, all-WT dimers dominate the species detected, with only a minor population (∼18% of the total ion intensity) of mixed WT/I26P dimers, and no evidence of I26P/I26P homodimers, consistent with the results obtained for I26P in isolation (Fig. 2f & 4b(i)). The conformational properties of the mixed WT/I26P dimer is indistinguishable from that of the all-WT species, measured by their ATDs (Fig. 4b(ii)). Thus, while I26P is refractory to homo-polymerization, it is capable of co-polymerization with more amyloidogenic sequences.

### The most rapidly assembling sequence determines the aggregation rate of peptide mixtures

To determine the impact of co-polymerization on the lag time of fibril assembly ThT profiles of 1 : 1 mixtures of the peptides were studied. Dramatic differences in the lag time of assembly were observed for the mixed samples (Fig. 5a–f(i) and ESI S5†). Amyloid fibrils were formed in all peptide mixtures over the time course of the experiment, as observed by TEM, including for the initially non-amyloidogenic Rat IAPP (Fig. 5a–f(ii)). Remarkably and unexpectedly, the lag-time of every peptide mixture was found to be determined by the lag time of the most amyloid-prone sequence alone in the mixture (*i.e.* the fastest aggregating sequence) (Fig. 5 & ESI Table 1†). For example, all 1 : 1 pairwise mixtures containing S20G formed amyloid instantaneously (Fig. 5f(iii)), with no inhibitory effects of less amyloid-prone variants observed. Similarly, 5 of the 6 samples containing H18L formed amyloid without a measurable lag phase, comparable to the rate of assembly of H18L alone, while H18L and S20G formed ThT positive aggregates immediately, consistent with S20G alone (Fig. 5d(i)). Similarly, the lag time of FreeCT variant is increased when mixed with the more rapidly assembling H18L or S20G peptides, with the lag time of these mixtures being indistinguishable from those of H18L or S20G alone (Fig. 5c(iii)).

FreeCT decreases the lag time of the more slowly aggregating I26P and the non-amyloidogenic Rat IAPP variants (Fig. 5b and e(i)), such that these peptides now assemble with the lag time of FreeCT alone (Fig. 5b and e(iii)). In the case of I26P, the lag time is decreased by all other sequences, with the slowest assembling mixture being I26P/Rat (Fig. 5e(iii)). Previous reports using 2D-IR and ESI-IMS-MS have suggested that the ability of WT and Rat IAPP to form hetero-oligomers similar to those formed from WT alone may rationalize why Rat IAPP is inefficient at inhibiting WT when the peptides are mixed at an equimolar ratio.^9,21^ Consistent with these reports, under the conditions used here Rat IAPP is also unable to inhibit assembly of all other amyloidogenic IAPP variants studied (Fig. 5b).

Whilst the behavior of these peptide mixtures may depend on the solution conditions employed (and indeed different behavior has been observed for IAPP using different pH, ionic strength, and molar ratios of different peptide sequences^42,46,62^), it is striking that inhibition is not observed in any of the peptide mixtures analyzed. Instead, in every case, the most amyloid-prone sequence determines the lag time of assembly. This observation is consistent with the behavior ascribed to prions, in which the conformational properties of a more amyloidogenic species can be transplanted onto compatible, but less amyloidogenic variants by a mechanism known as conformational templating.^63–66^ Consistent with this phenomenon occurring for IAPP under the conditions employed here, ESI-IMS-MS directly shows an increased population of more expanded species (dimers and trimers with long arrival times) which correlate with a decrease in lag time for H18L/WT (Fig. 4 and ESI S4†). In addition, the lack of biphasic assembly kinetics visualized by ThT fluorescence (Fig. 5, ESI 5†) provides strong evidence for co-assembly, rather than independent aggregation, of the different peptides in early stages of aggregation.

To determine the contribution of each peptide sequence to the amyloid products formed, the aggregated mixed samples were collected using ultracentrifugation and the pellets depolymerized by incubation in 100% HFIP for 24 h, with agitation. Samples were then air-dried and re-suspended in denaturing solvent (50 : 40 : 10 (v/v/v) acetonitrile/water/acetic acid (Methods)). The resulting ESI mass spectra (ESI Fig. 6†) showed the presence of both precursor sequences in the pellet for each mixed sample. The relative abundance of peptide in the aggregated phase of the different sequences is approximately proportional to the amyloid propensity (as judged by the length of the lag phase). For example, approximately twice as many H18L monomers are incorporated into fibrils compared with Rat IAPP monomers in 1 : 1 mixtures of the two sequences. All monomer sequences were also found in the supernatant of each mixed sample, with the least amyloid prone monomers being in excess here (ESI Fig. 6†), in agreement with previous observations for mixtures of WT and Rat IAPP.^9^ These results cannot determine whether individual fibrils contain peptide mixtures, or if the different sequences ultimately form homo-polymeric fibrils subsequent to mixing in the early stages of assembly. Nonetheless, the results presented show unequivocally that IAPP sequences that form amyloid rapidly are able to accelerate amyloid formation of more slowly aggregating sequences by co-assembly in the lag time of assembly.

### Peptide variants that co-polymerize do not cross-seed

To determine whether the variants are able to cross-seed fibril formation of one another, WT and S20G fibrils formed from their respective pure peptides were used to seed solutions of WT, S20G or Rat IAPP at a ratio of 10% (w/w) seeds. The resulting ThT profiles (ESI Fig. 7a and c†) showed that WT fibrils are able to seed fibril formation of WT monomer, but do not cross-seed fibril formation of Rat. Similarly, S20G fibrils are not able to cross-seed WT or Rat IAPP fibril formation. The effect of seeding in the samples containing S20G monomer could not be determined given that fibrillation occurs instantaneously even in the absence of seeds (ESI Fig. 7b†). Most importantly, the results demonstrate that the effects of peptide mixtures on the lag phase of assembly cannot be explained by cross-seeding, at least for the sequences studied here.

## Conclusions

There is an urgent need to develop therapies against amyloid disease. To date only a single therapy – tafamidis^67^ which stabilizes the native tetramer of transthyretin and prevents its aggregation – is in the clinic. What is needed is a greater understanding of the species formed during aggregation and identification of the toxic agent(s) of disease. Indeed, both pre-fibrillar oligomers^22,68,69^ and mature fibrils have been shown to induce cytotoxicity in different amyloid proteins.^70–73^ In the case of IAPP, amyloid formation is widely considered to be a significant factor in the deterioration of islet function and reduction in beta cell mass,^19^ which contributes to type II diabetes mellitus. Indeed, amyloid plaque load has been demonstrated to be proportional to disease progression.^74^ The physio-chemical properties of the toxic species produced during islet amyloidosis, however, remain elusive. Identifying and characterizing the oligomeric species formed by these self-assembling proteins is key to unravelling this mystery. Our manuscript presents this much-needed information: using ESI-IMS-MS to identify and characterize the oligomers formed by IAPP and ascribing the population of specific oligomeric conformers to the lag time of amyloid formation. Such information will help to define the nature of amyloid-associated toxicity and may pave the way towards therapies in the long term.

Co-polymerization of variant protein sequences is known to occur in amyloid formation *in vivo* with the relative concentration of different sequences or unmodified/post-translationally modified sequences being important indicators of the threat of disease.^30,75,76^ Despite the importance of co-polymerization in amyloid assembly, we are only now beginning to understand how mixed sequences can alter the course of aggregation in mechanistic detail, for example by cross-seeding (in which fibril fragments of a highly amyloidogenic protein can enhance polymerization of less amyloidogenic variants).^56,59,77–79^ In addition to sequence variants, amyloid deposits can also contain glycosaminoglycans, nucleic acids, serum amyloid P and other co-factors, which add to the complexity of amyloid assembly *in vivo* ^80,81^ and can modulate the rate of assembly by altering the potentials for secondary nucleation and/or fragmentation.^66,80,82,83^ Enhancing the probability of assembly by templating an amyloidogenic conformation onto a previously innocuous protein homologue, as shown famously for prions, provides a third mechanism by which co-assembly can enhance aggregation potential.^7,31,77,84^ Determining how mixing protein sequences can affect oligomer formation in the initiating events in assembly is even more challenging, especially for initially intrinsically disordered peptides, such as Aβ, α-synuclein and IAPP, because of the difficultly of studying each participating sequence in the heterogeneous mixtures of transiently populated oligomers that form in the lag time.

Here, by combining ThT fluorescence analyses with the powers of ESI-IMS-MS we were able to identify and characterize monomers and oligomers of IAPP in individual sequences and in pairwise mixtures and demonstrate that co-assembly into hetero-oligomers can have a dramatic effect on the lag time of amyloid formation, with the most aggregation-prone IAPP sequences able to accelerate amyloid formation of their less aggregation-prone counterparts in a mechanism reminiscent of that of prions. Indeed, using ESI-IMS-MS we show that the conformational ensemble of mixed oligomers formed during co-polymerization of H18L/WT is altered compared with their homo-oligomeric counterparts, with the population of the most expanded hetero-dimers and trimers correlating with a decreased lag time of assembly. We also show that the requirements for cross-seeding are stricter than those for co-polymerization of the IAPP sequences studied here, with sequences able to co-polymerize (*e.g.* S20G/WT or Rat/WT) being unable to seed elongation of each other. Cross-seeding requires structural compatibility, so that monomers or oligomers of one sequence can recognize the cross-β surface presented by the seed formed from the second sequence. By contrast, co-polymerization of different sequences into oligomers has less strict steric requirements, with different sequences able to co-assemble and to alter each other's conformational properties. Similar results have been observed for WT IAPP and Aβ40, with mixing resulting in co-polymerization,^32^ but seeding being more complex, with Aβ40 fibrils being able to cross-seed hIAPP monomers, but not *vice versa*.^10,85,86^ The differences are reminiscent of the classic lock and key *versus* induced fit models of binding. The ESI-IMS-MS studies also reveal a striking relationship between the conformational properties of monomeric IAPP and the length of the lag phase. These results show that increased population of the more expanded conformer detected by ESI-IMS-MS results in a shorter lag time, ascribing the most expanded conformer as the most amyloidogenic species: information that has previously remained elusive and which could be important for the rational design of anti-amyloid agents.

Co-polymerization has been shown to occur in other systems and to have different effects on the course of amyloid formation both *in vitro* and *in vivo*, with some sequences able to stimulate assembly, whilst others inhibit aggregation, dependent on the precise nature of the proteins involved and the solution conditions employed. For example, mixing monomers of human and murine transthyretin (TTR), a folded protein, results in the formation of stable, mixed tetramers which abolish fibril formation.^87^ Similarly, stabilization of TTR hetero-tetramers achieved by mixing the aggregation-protective T119M variant with the amyloid-prone V30M variant protects heterozygotes from disease.^88^ For β_2_-microglobulin, which aggregates into amyloid *via* a partially folded monomer,^59^ mixing the human protein with non-amyloidogenic murine β_2_m retards assembly *via* formation of specific heterodimers, whilst combining the human protein with the more amyloidogenic truncated variant, ΔN6, accelerates its aggregation.^35^ For the intrinsically disordered proteins, Aβ and α-synuclein, mixing can also alter the course of assembly: Aβ42 and Aβ40 can co-assemble,^89^ however Aβ40 retards the aggregation of Aβ42 in a concentration-dependent manner,^90^ while the E22G (Arctic mutation) of Aβ40 arrests assembly of WT Aβ40 at the protofilament stage.^91^ Similarly, β-synuclein and γ-synuclein fail to form co-polymers with,^92^ but can inhibit the assembly of, α-synuclein.^93,94^ Understanding how different protein sequences are able to alter the course of assembly, such as demonstrated here for IAPP, will help to shed light on the fundamental molecular mechanisms of amyloid formation, as well as the biological consequences of amyloid deposition and the aetiology of disease. Combining the powers of kinetic analyses of sequence variants with the ability of ESI-IMS-MS to identify, quantify and characterize rare, transient and rapidly interconverting species offers unique potential to interrogate the very earliest events in amyloid assembly and to better understand the sequence and structural requirements of prion-like behavior, conformational templating, co-polymerization and cross-seeding.
