# Supplementary material for: Understanding co-polymerization in amyloid formation by direct observation of mixed oligomers
Source: Chem Sci. 2017 May 9;8(7):5030–40. doi: 10.1039/c7sc00620a (PMC5613229; doi:10.1039/c7sc00620a)
Supplement: Supplementary file 1 [file SC-008-C7SC00620A-s001.pdf]

Electronic Supplementary Material (ESI) for Chemical Science.
This journal is © The Royal Society of Chemistry 2017

Supplementary Information

Understanding co-polymerization in amyloid formation by direct observation of mixed oligomers

Lydia M. Young,[a] Ling-Hsien Tu,[b,c] Daniel P. Raleigh,[b] Alison E. Ashcroft[a] and Sheena E. Radford*[a]

[a] Astbury Centre for Structural Molecular Biology, School of Molecular and Cellular Biology, University of Leeds, Leeds LS2 9JT, U.K. [b] Department of Chemistry, Stony Brook University, Stony Brook, New York 11794-3400, USA. [c] Genomics Research Center, Academia Sinica, 128 Academia Taipei 11529, Taiwan

Corresponding Author

* E-mail: s.e.radford@leeds.ac.uk

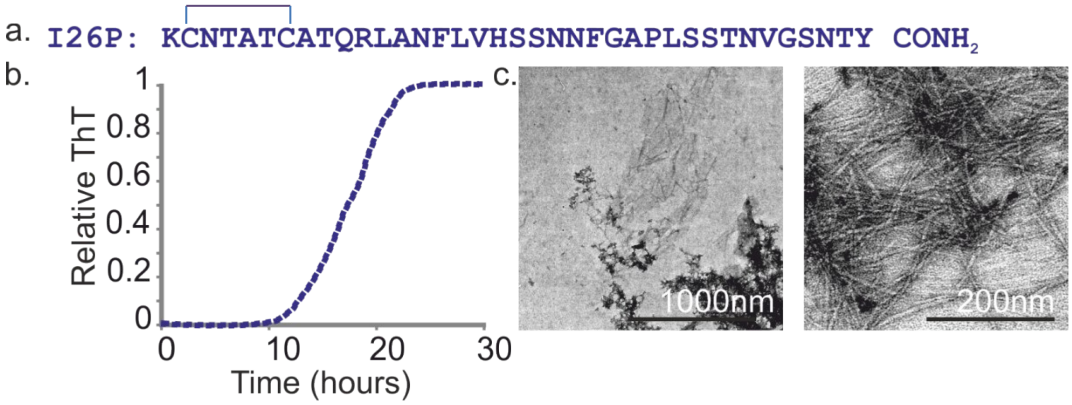

Figure S1. **Sequence and aggregation of the IAPP variant, I26P.** *a) Primary sequence I26P. b) ThT fluorescence intensity experiments of I26P (32 μM peptide, 25 °C, quiescent) in a 1:1 (v/v) mixture of 100 mM ammonium acetate: 100 mM ammonium bicarbonate, 1% (v/v) DMSO, pH 7.4. Representative negative stain TEM images show I26P aggregate (left image) and some fibrils (right image) after 5 days incubation under the same conditions (scale bar = 1000 nm or 200 nm, as indicated).*

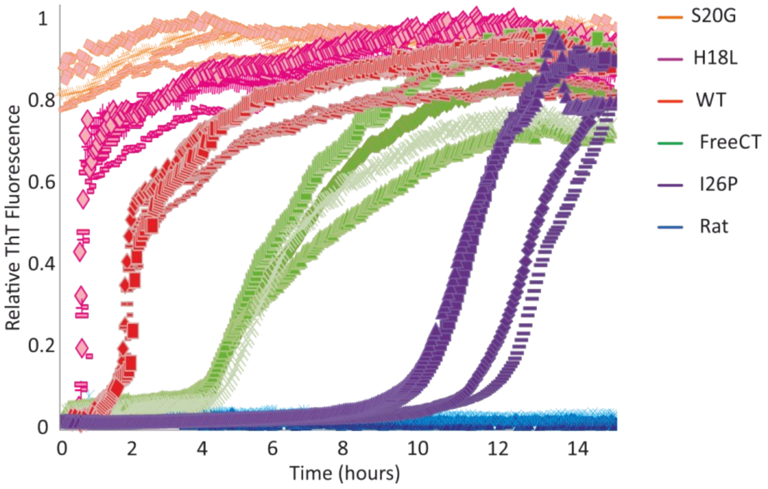

Figure S2. **Different IAPP sequences aggregate into amyloid fibrils with different kinetics**. *ThT fluorescence intensity experiments of different peptides (32 µM peptide, 25 °C, quiescent) in a 1:1 (v/v) mixture of 100 mM ammonium acetate: 100 mM ammonium bicarbonate, 1% (v/v) DMSO, pH 7.4, show varying lag times. Four replicates of each sequence are shown, acquired in the same plate.*

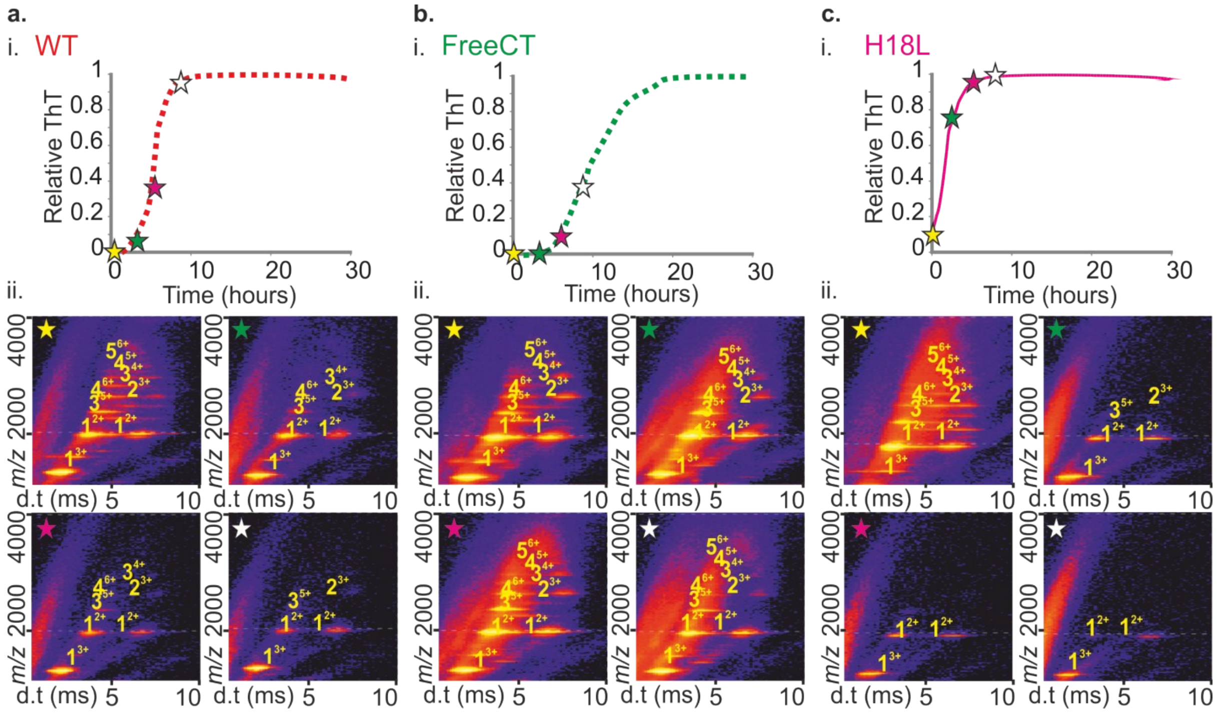

Figure S3. **Dependence of oligomer distributions of different IAPP sequences on the rate of fibril formation.** *ThT fluorescence intensity of a) i. WT, b) i. Free-CT and c) i. H18L (32 μM peptide, 25 °C, quiescent). ESI-IMS-MS driftscope plots of a) ii. WT, b) ii. Free-CT and c) ii. H18L oligomers present at t = 2 min (yellow star), t = 2.5 hours (green star), t = 5 hours (pink star), t = 7.5 hours (white star). d.t. = drift time.*

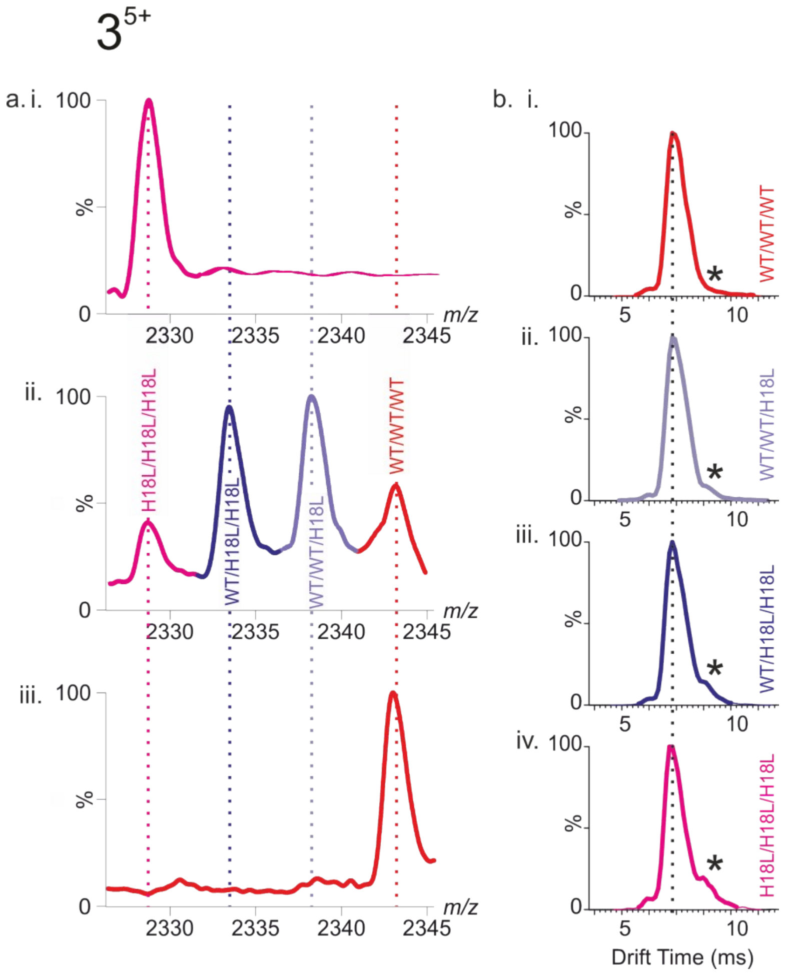

Figure S4. **Mixing WT and H18L IAPP reveals random assembly of the different peptide sequences into trimers**. *a) ESI mass spectra of H18L alone (i), a 1:1 mixture of WT:H18L (ii) and WT alone (iii), showing the presence of both homo- and hetero-trimers carrying five charges. b) Arrival time distributions of homo- (i and iv) and hetero-trimers (ii and iii). Each trimer occupies one dominant conformeric ensemble with a small contribution of a second more expanded conformer (*) which increases in relative intensity as the number of H18L monomer units increases. Note, data for WT/H18L are shown because this effect is observed most easily for the variants that have mass differences that allow all homo- and hetero-oligomeric peaks to be resolved.*

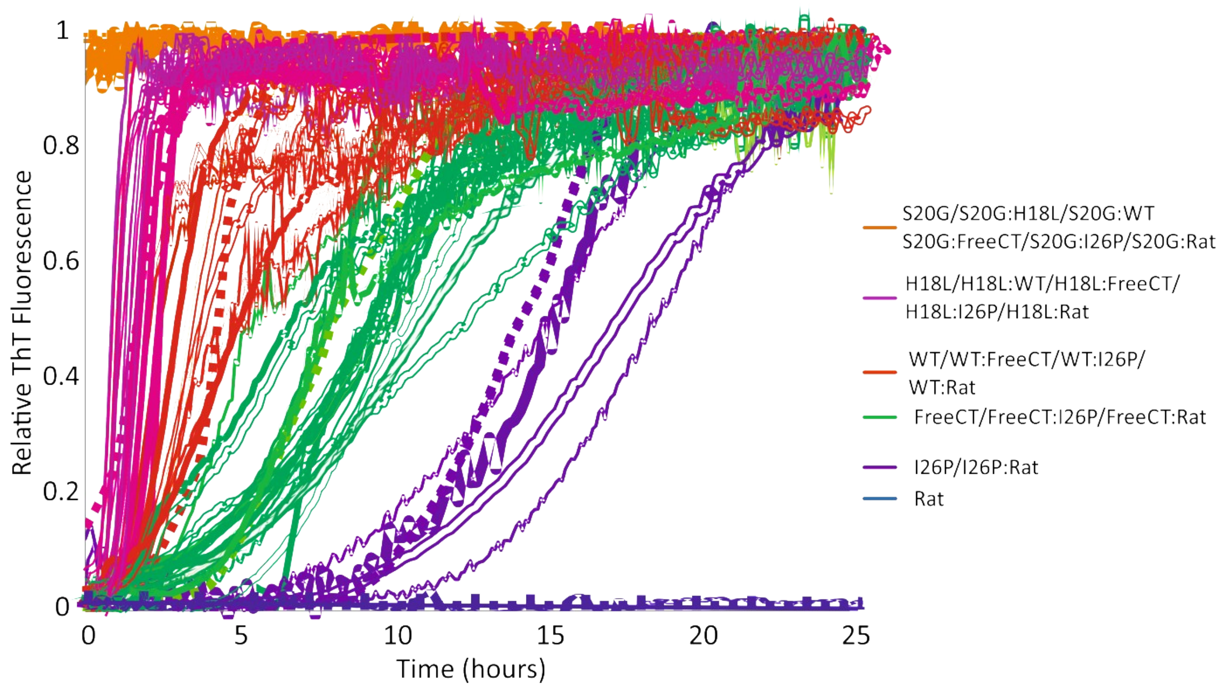

Figure S5. ThT fluorescence intensity experiments following amyloid formation from IAPP sequence variants as well as 1:1 mixtures of different peptide sequences. *ThT experiments (32 μM final peptide concentration, 25 °C, quiescent) in a 1:1 (v/v) mixture of 100 mM ammonium acetate: 100 mM ammonium bicarbonate, 1% (v/v) DMSO, pH 7.4) for every sequence and mixture were repeated a minimum of three times on each plate, all data from a single plate are shown here. The average of each set of curves is shown as a dotted line.*

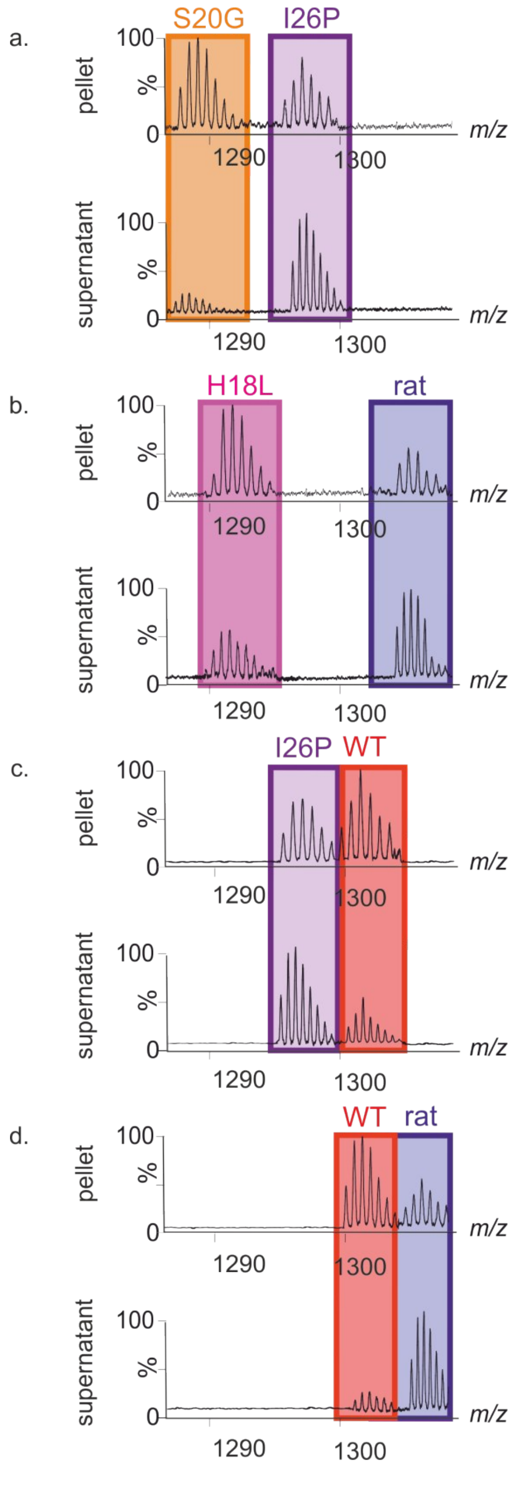

Figure S6. **ESI mass spectra of depolymerized fibrils formed from mixtures of peptide sequences show the presence of both monomer sequences.** *The pellet and supernatant of each sample were collected by ultracentrifugation, depolymerized using HFIP and the presence or absence of each sequence in each fraction was assayed using ESI-MS: a) S20G and I26P, b) H18L and Rat, c) I26P and WT, d) WT Rat. The 3+ charge state is shown.*

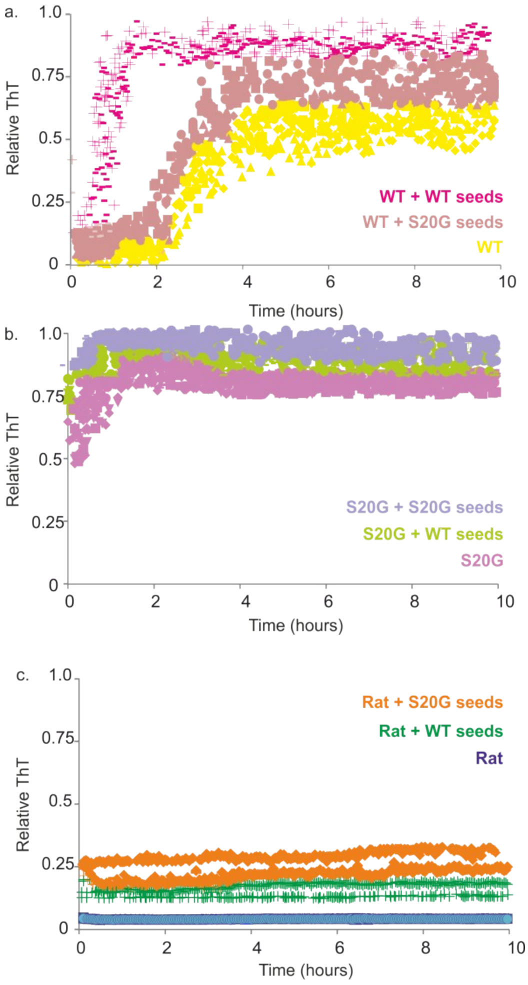

Figure S7. **Peptide sequences that co-assemble do not cross-seed.** *ThT fluorescence intensity experiments of a) WT, b) S20G and c) Rat IAPP (32 μM peptide, 25 °C, quiescent) with or without 3.2 μM (monomer concentration) of WT or S20G seeds. The fluorescence intensity of the mixture of Rat IAPP with the WT and S20G seeds is slightly higher than that of the Rat peptide alone due to the fact that seeds bind to ThT. All experiments were performed in a 1:1 (v/v) mixture of 100 mM ammonium acetate: 100 mM ammonium bicarbonate, 1% (v/v) DMSO, pH 7.4.*

Sample	Average lag time (hours)
WT/S20G	0.0
I26P/S20G	0.0
S20G/Rat	0.0
S20G/H18L	0.0
S20G	0.0
S20G/FreeCT	0.0
H18L/Rat	0.3
H18L/FreeCT	0.3
WT/H18L	0.3
H18L	0.4
H18L /I26P	0.5
WT/FreeCT	2.0
WT	2.2
WT/Rat	2.4
WT/I26P	2.7
I26P/FreeCT	4.2
Rat/FreeCT	4.4
FreeCT	4.5
Rat/I26P	7.1
I26P	9.8
Rat	∞

Table S1 Average lag times from ThT fluorescence intensity experiments of individual sequences and 1:1 mixtures of different peptide sequences. *A minimum of three replicates of each sample was used in calculating average lag times (32 μM each peptide, 25 °C, quiescent) in a 1:1 (v/v) mixture of 100 mM ammonium acetate: 100 mM ammonium bicarbonate, 1% (v/v) DMSO, pH 7.4.). Error on the average lag time is +/- 10% on a single plate. Day to day variation in lag times is greater. However the effect of peptide mixtures observed is highly reproducible, irrespective of the absolute length of the lag phase.*
